# Treatment selection of early stage non-small cell lung cancer: the role of the patient in clinical decision making

**DOI:** 10.1186/s12885-018-3986-5

**Published:** 2018-01-15

**Authors:** S. Mokhles, J. J. M. E. Nuyttens, M. de Mol, J. G. J. V. Aerts, A. P. W. M. Maat, Ö. Birim, A. J. J. C. Bogers, J. J. M. Takkenberg

**Affiliations:** 1 Department of Cardio-thoracic Surgery, Erasmus-MC, Room Bd-577, P.O. Box 2040, 3000 CA Rotterdam, The Netherlands; 2 Department of Radiation Oncology, Erasmus-MC-Cancer Institute, Rotterdam, The Netherlands; 3 Department of Pulmonary Disease, Erasmus-MC, Rotterdam, The Netherlands; 4 Department of Pulmonary Disease, Amphia Hospital, Breda, The Netherlands

**Keywords:** Cancer patients, Decision-making preferences, Shared decision-making, Surgery, Radiation oncology

## Abstract

**Background:**

The objective of this study is to investigate the role and experience of early stage non-small cell lung cancer (NSCLC) patient in decision making process concerning treatment selection in the current clinical practice.

**Methods:**

Stage I-II NSCLC patients (surgery 55 patients, SBRT 29 patients, median age 68) were included in this prospective study and completed a questionnaire that explored: (1) perceived patient knowledge of the advantages and disadvantages of the treatment options, (2) experience with current clinical decision making, and (3) the information that the patient reported to have received from their treating physician. This was assessed by multiple-choice, 1–5 Likert Scale, and open questions. The Decisional Conflict Scale was used to assess the decisional conflict. Health related quality of life (HRQoL) was measured with SF-36 questionnaire.

**Results:**

In 19% of patients, there was self-reported perceived lack of knowledge about the advantages and disadvantages of the treatment options. Seventy-four percent of patients felt that they were sufficiently involved in decision-making by their physician, and 81% found it important to be involved in decision making. Forty percent experienced decisional conflict, and one-in-five patients to such an extent that it made them feel unsure about the decision. Subscores with regard to feeling uninformed and on uncertainty, contributed the most to decisional conflict, as 36% felt uninformed and 17% of patients were not satisfied with their decision. HRQoL was not influenced by patient experience with decision-making or patient preferences for shared decision making.

**Conclusions:**

Dutch early-stage NSCLC patients find it important to be involved in treatment decision making. Yet a substantial proportion experiences decisional conflict and feels uninformed. Better patient information and/or involvement in treatment-decision-making is needed in order to improve patient knowledge and hopefully reduce decisional conflict.

**Electronic supplementary material:**

The online version of this article (10.1186/s12885-018-3986-5) contains supplementary material, which is available to authorized users.

## Background

Surgical resection is considered the preferred treatment for patients with early-stage non-small cell lung cancer (NSCLC). A less invasive option for patients with comorbidities is stereotactic body radiotherapy (SBRT) [1, 2]. Several studies have demonstrated that SBRT may be as effective as surgery in potentially operable patients, however, randomized trials with larger patient populations and longer follow-up are still lacking [3–5]. In this setting it is important to provide adequate information to allow patients to take an active role in treatment decision.

Shared decision making (SDM) is a process in which physician and patient work together in making a health decision after discussing the options, the benefits and harms, and considering the patients’ values, preferences, and circumstances [6, 7]. SDM is seen as the middle ground between informed choice, where the patient makes the decision based on information received from the physician, and traditional paternalistic decision making, where the physician makes the decision based on best available evidence [8, 9]. Patients who are active participants in the process of their care, for example asking questions, expressing their opinions and preferences, have better health outcomes, more knowledge regarding the disease and they are less anxious than patients who do not participate in the decision making [7, 10–12]. SDM supports patient to understand the disease and weigh advantages and disadvantages of treatment options in their own context, which will result in an informed treatment decision making with patients’ needs and values incorporated. Although SDM has gained increased awareness among the healthcare community, it has not been widely incorporated into routine clinical practice in lung cancer care. This can be explained by the fact that there is lack of familiarity with SDM [13, 14], and also because the care of lung cancer patient can be complex due to multiple treatment types over an extended period of time and often includes a guideline-drive treatment [15]. Furthermore, there are a number of factors that complicate the implementation of SDM in current clinical practice such as guideline based treatments, patient knowledge, time constrains and care settings [16, 17].

This study assesses among Dutch early-stage NSCLC patients: (1) perceived patient knowledge of the advantages and disadvantages of treatment options, (2) experience with current clinical decision-making, and (3) perceived understanding of information regarding their disease and the treatment.

## Methods

### Patient population

Between December 2012 and December 2014, 155 consecutive patients with stage I or II NSCLC were recruited for this prospective observational study. These patients were subsequently treated surgically or with SBRT at Erasmus University Medical Center, Erasmus MC-Cancer Institute, or Amphia Hospital Breda. Consecutive patients were contacted by telephone to explain the purpose of the study and obtain their consent to receive a questionnaire. Only patients who agreed to participate and provided written informed consent were eligible for the inclusion in this study (*n* = 84). The overall response rate was 54%. No significant differences were found between responders and non-responders in terms of baseline characteristics. This study was approved by the institutional review board of Erasmus University Medical Center (MEC 2012-462).

Clinical staging of patients treated surgically (*n* = 55) or with SBRT (*n* = 29) was done with CT-scan, ^18^FDG-PET imaging and/or using (minimally invasive) endoscopic techniques when appropriate. Clinical and pathological staging was based on American-Joint-Committee-in-Cancer 7th-edition staging manual [18]. Chronic obstructive pulmonary disease (COPD) was defined according to the GOLD criteria [19]. Comorbidity-scores were recorded using the Charlson-Comorbidity-Index (CCI) [20]. Treatment planning of patients who received SBRT have been described previously [21]. All patients were discussed in a multidisciplinary team meeting before being accepted for treatment.

### Data collection

Baseline characteristics of patients were collected by reviewing the patients’ medical records and hospital information system. After the treatment decision was made but before the actual start of the treatment, patients completed a questionnaire. The aim of this questionnaire is to investigate: (1) perceived patient knowledge of the advantages and disadvantages of treatment options, (2) experience with current clinical decision-making (this includes the preferences, patient experience and involvement in treatment decision-making using Decisional Conflict Scale (DCS) and Control Preferences Scale (CPS), and (3) perceived understanding of information regarding their disease and the treatment. These components are measured at baseline using multiple-choice questions, a 1–5 Likert Scale, and open questions. Health-related-quality-of-life (HRQoL) was measured before the treatment, 6 months and 12 months after the treatment using the Short-Form 36-Item Health Survey (SF-36). For details regarding the questionnaire see Additional file 1.

#### Control preference scale

The patients’ preferred decisional role was assessed using a modified version of the CPS. The CPS is an instrument that assesses preferences regarding patient participation in health care decisions. Patients were asked to select one of the five statements on roles in treatment decision-making; (A) the physician makes the decision about the treatment alone, (B) the physician makes the decision after considering the patient’s opinion, (C) the patient makes the decision together with the clinician, (D) the patient makes the decision after considering the doctor’s opinion, and (E) the patient makes the decision about the treatment alone [22–24]. This scale has been widely used in previous studies [25, 26]. To investigate the potential association between education level and CPS patients were asked to indicate their educational attainment.

#### Decisional conflict scale

The DCS was used to assess the level of ‘decisional conflict’ that patients experience while making health care decisions. This scale has been extensively validated and has been widely used. The DCS measures decision uncertainty that leads to decision delay, and quantifies modifiable factors which contribute to uncertainty. It contains 16 items, each using a five-point Likert response format (i.e. completely agree, agree, neither agree nor disagree, disagree, completely disagree). These items are combined to form total score and five subscales (i.e. uncertainty, informed, values clarity, support, and effective decision subscore). Scores lower than 25 are associated with implementing decisions and scores exceeding 37.5 are associated with delay or feeling unsure about implementation [27, 28]. In case of missing values (<6%) we used a multiple imputation technique to impute missing values in order to avoid them being depicted as ‘unknown’ in incomplete observations. We have used 5-fold multiple imputation using SPSS for Windows version 21 [29]. In the surgery group 32 and 19 patients were alive at 6 and 12 months without tumor progression, respectively. In the SBRT group this was 9 and 4 patients at 6 and 12 months, respectively. Due to the low response rates at 6 and 12 months we could not explore decisional conflict over time.

#### Health related quality of life assessment

HRQoL was measured with the SF-36. The SF-36 is the most extensively used and evaluated health outcomes measure and has shown to be valid and reliable in multiple populations. The SF-36 assess eight self-reported aspects of HRQoL (i.e. physical functioning, role physical functioning, role emotional functioning, mental health, vitality, social functioning, bodily pain, and general health). It also yields physical (PCS) and mental (MCS) health summary measures. Scale scores are obtained by summing the items together within a domain, dividing this outcome by the range of scores and then transforming the scores to a scale from 0 to 100 [30]. The mean score of the PCS and MCS is 50 with a standard deviation of 10 and wherein a higher score means a better health status. Furthermore, a higher score on the SF-36 subdomains represents a better functioning; a high score on the bodily pain scale indicates the absence of pain. The scale has good reliability, with Cronbach α ranging from 0.65 to 0.96 for all subscales [31]. We used the Dutch adaptation of the SF-36 health status scale [32]. Patients were asked to complete the SF-36 form after treatment decision was made but before the treatment (baseline), at 6 and 12 months to all surviving patients. In case of missing values we applied simple imputation [33, 34]. HRQoL was assessed in 84 patients at baseline (surgery = 55, SBRT = 29). Due to the low response rates at 6 and 12 months (surgery group 32 and 19 patients were alive at 6 and 12 months and this was in the SBRT group 9 and 4 patients, respectively) the effect of time could not be analyzed.

Local control and the presence of metastases were defined according to the guidelines of ACCP and STS [35]. Twelve patients were diagnosed with tumor recurrence after the treatment, four of these patients had both loco-regional and distant recurrence.

### Statistical analysis

Continuous data are reported as mean ± SD or median with range, and categorical data are reported as proportions. Normally distributed continuous variables were compared by using Student *t* tests, and not normally distributed (Kolmogorov-Smirnov) data were compared by using the Mann-Whitney-U-test. Discrete variables were compared by using the Chi-Square test or the Fisher Exact test where appropriate. Aim 1 and 3 of this manuscript were analyzed using simple statistics by counting the ‘yes’ and ‘no’ answers. Components measured with 1–5 Likert-scale were not categorized.

A general linear model (GLM) with the bootstrap method was used to assess the association between HRQoL measured at baseline and 1) patient experience with involvement in treatment selection, 2) patient preferences for SDM, and 3) patients’ preferred decisional role in treatment decision-making (assessed with CPS). The purpose behind the use of bootstrapping is to account for skewed distribution of residuals of SF-36 variables [36, 37] and to obtain valid and reliable *p*-values.

All statistical tests were two-tailed and a *p*-value of <0.05 was regarded as statistical significant. The statistical software package SPSS for Windows version 21 (SPSS Inc., Chicago, IL) was used for data analysis. GraphPad Prism5.00 for Windows (GraphPad software, San Diego, CA) was used to obtain graphs of QoL.

## Results

The baseline characteristics of all 84 patients are listed in Table 1. In 55 patients surgical treatment was chosen (median age = 65), in 29 patients SBRT (median age = 73). In this cohort of patients the education level was in accordance with the education level of the general Dutch population [38].

**Table 1:** Patient characteristics

Characteristics	Total (*n* = 84)	Surgery (*n* = 55)	Radiotherapy (*n* = 29)	*P-value*
Sex				*0.406*
-Male (%)	44 (52)	27 (49)	17 (59)	
-Female (%)	40 (48)	28 (51)	12 (41)	
Age, median (range)	68 (50–87)	65 (50–81)	73 (52–87)	*0.001*
Education level (%):				*0.875*
-Primary education	12 (14)	8 (15)	4 (14)	
-Secondary education	21 (55)	29 (53)	17 (59)	
-Higher education	46 (27)	15 (27)	8 (27)	
-Other	3 (4)	3 (5)	–	
Smoking habits				
-Nonsmoker (%)	3 (4)	2 (4)	1 (3)	*0.588*
-Current or former smoker (%)	60 (71)	38 (69)	22 (76)	
-Unknown, n (%)	21 (25)	15 (27)	6 (21)	
FEV_1_% mean ± SD^a^	80 (24)	87 (20)	67 (26)	*0.001*
-Unknown, n (%)	3 (4)	2 (4)	1 (3)	
DLCO (%) mean ± SD^b^	76 (24)	83 (22)	61 (22)	*<0.001*
COPD (%)^c^				*0.001*
-No COPD	38 (45)	31 (56)	7 (24)	
-GOLD I	17 (20)	10 (18)	7 (24)	
-GOLD II	19 (23)	13 (24)	6 (21)	
-GOLD III	8 (10)	1 (2)	7 (24)	
-GOLD IV	2 (2)	–	2 (7)	
Charlson comorbidity index (%)				*0.026*
- ≤ 1	47 (56)	33 (60)	14 (48)	
-2–3	26 (31)	17 (31)	9 (32)	
-4	6 (7)	3 (5)	3 (10)	
- ≥ 5	5 (6)	2 (4)	3 (10)	
Clinical stage (%)				*0.001*
-IA	47 (56)	22 (40)	25 (86)	
-IB	14 (17)	12 (22)	2 (7)	
-IIA	17 (20)	15 (27)	2 (7)	
-IIB	6 (7)	6 (11)		
Pathological stage (%)				
-IA	17 (31)	17 (31)	–	
-IB	18 (33)	18 (33)	–	
-IIA	9 (16)	9 (16)	–	
-IIB	7 (13)	7 (13)	–	
-IIIA/B	4 (7)	4 (7)	–	
Histology (%)				*0.262*
-Squamous cell carcinoma	18 (21)	14 (26)	4 (14)	
-Adenocarcinoma	21 (25)	15 (27)	6 (21)	
-Large cell carcinoma	8 (10)	6 (11)	2 (7)	
-NSCLC	37 (44)	20 (36)	17 (58)	
Clinical tumor diameter (mm), median (range)	25 (7–130)	29 (7–130)	22 (9–41)	*<0.001*
-Unknown, n (%)		11 (5)	–	
Pathological tumor diameter (mm), median (range)	28 (1–90)	28 (1–90)	–	

^a^FEV_1_%: Forced expiratory volume in 1 s expressed as a percent of predicted

^b^Diffusion capacity of the lung for carbon monoxide

^c^COPD: chronic obstructive pulmonary disease

### Perceived patient knowledge regarding the treatment

Self- reported lack of knowledge about the advantages and disadvantages of the treatment options was present in 18% of patients in the surgery group and in 22% of patients in the SBRT group. Self-reported lack of knowledge about the treatment risks was present in 6% of patients in the surgery group and in 21% of patients in the SBRT group.

### Experience with current clinical decision-making

#### Patient preferences for SDM

The majority (85%) of patients agreed that ideally decision-making should be done together with the physician. Twelve percent of patients wanted to leave the decision about the appropriate treatment to their treating physician and 3% indicated that the decision should be done mainly by patients. No association was found between the education level and the control preference scale.

#### Experience in treatment decision-making

On average, patients in this cohort discussed their treatment with three physicians. The majority of patients in the surgery and SBRT group involved a family member in making the choice for a treatment, 75 and 68%, respectively. Most of patients thought that they had enough time to make an informed decision (80% in the surgery group and 79% in the SBRT group). Patients indicated that several subjects were discussed during the conversation with their treating physician. Two percent of patients in the surgery group had the feeling that not every aspect of the treatment was discussed during the conversation with their treating physician. This was 11% in the SBRT group.

In the surgery group, 40% of patients experienced decisional conflict (score > 25), and 22% to such an extent that they felt unsure about their decision (score > 37.5). Thirty-two percent felt uncertain about the best choice, and 39% felt uninformed. Twenty-nine percent felt unclear about personal values for benefits and side effects of the treatment. Twenty-one percent felt unsupported in decision-making, and 21% of patients were not satisfied with their decision.

**Fig. 1:**
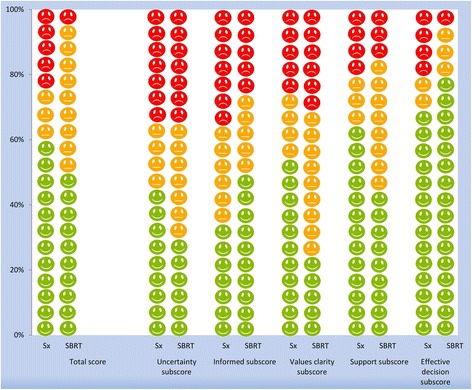
Decisional conflict in patients treated surgically or with stereotactic body radiotherapy (SBRT). Scores <25 (green smiley) are associated with implementing decisions and scores <37.5 (red smiley) are associated with delay or feeling unsure about implementation. Orange smiley represent scores between 25 and 37.5

In the SBRT group, 48% of patients experienced decisional conflict, and 7% to such an extent that they felt unsure about their decision. Thirty-five percent felt uncertain about the best choice, and 29% felt uninformed. Thirty-two percent felt unclear about personal values for benefits and side effects of the treatment. Fourteen percent felt unsupported in decision-making, and 7% of patients were not satisfied with their decision. Subscores on feeling uninformed and on uncertainty contributed the most to decisional conflict. Scores exceeding 37.5 are described here, details of the total score and five subscales for the two treatment groups are illustrated in Fig. 1.

#### Involvement in treatment decision-making

Seventy-four percent of patients felt that they were sufficiently involved in decision-making by their physician, 73% felt that they had a choice between different treatment options, 81% found it important to be involved in decision-making, 6% reported that alternative treatment options and complementary treatments were not discussed during the conversation about their treatment. Patients mentioned immunotherapy, diet and vitamin supplements as an example. Involvement in treatment decision-making for the two treatment groups can be found in Table 2.

**Table 2:** Involvement in treatment decision making for the two treatment groups

Involvement in decision making	Surgery (%)	Radiotherapy (%)
- Felt sufficiently involved	78	68
- Found important to be involved	78	89
- Having a choice	71	79
- Not having a choice	18	7

### Perceived understanding of information regarding the disease and the treatment

**Fig. 2:**
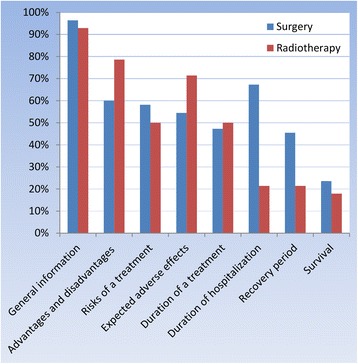
Information that the patient received during the consultation

Patients were asked to report which topics were discussed during the conversation about their treatment. Figure 2 illustrates that the minority of patients who undergone surgery or radiation therapy received information about the survival, 24 and 18%, respectively.

### Health related quality of life assessment

**Fig. 3:**
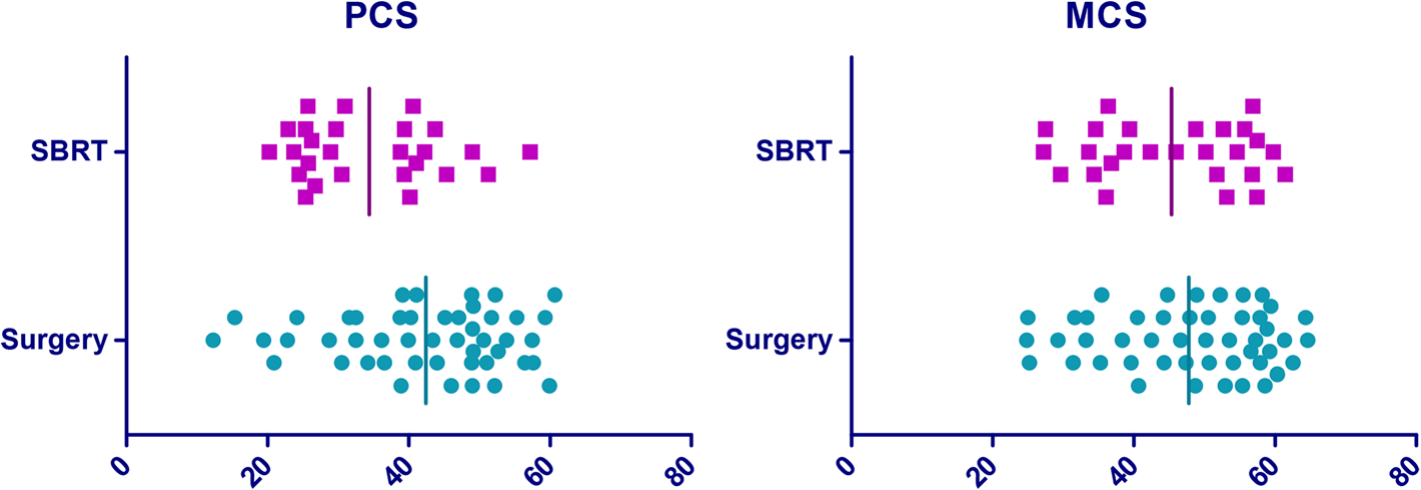
Scatterplot of physical component summary (PCS) and mental component summary (MCS) at baseline in the surgery and stereotactic body radiotherapy (SBRT) group

**Fig. 4:**
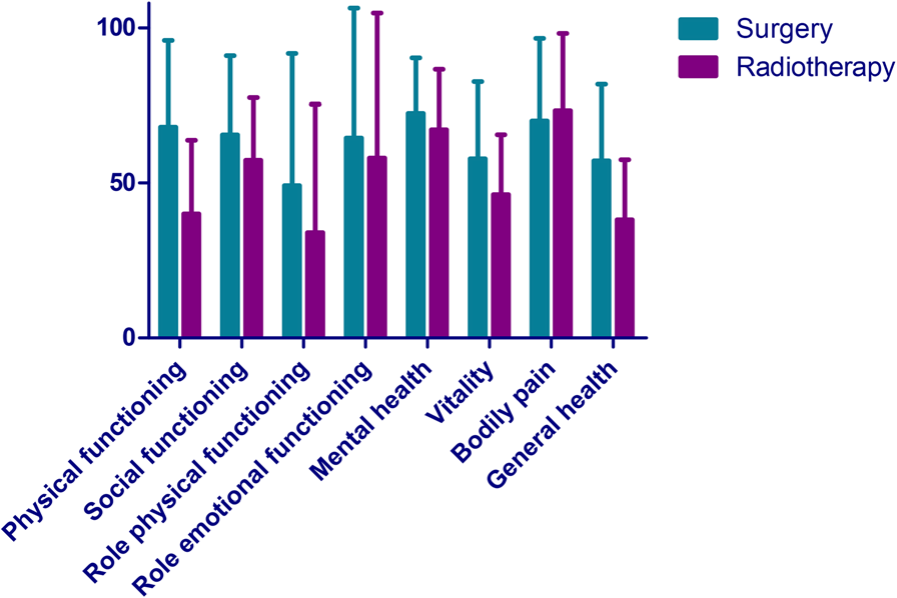
Eight self-reported aspects of HRQoL measured at baseline. The scores are expressed as the mean score with a standard deviation stratified by treatment group. A high score indicates better HRQoL, with a high score on bodily pain representing absence of pain

At baseline, patients in the surgery group scored higher on physical component summary (mean 42.4 ± 12.3) than patients in the SBRT group (mean 34.4 ± 10.1), Fig. 3. No major differences could be found between the HRQoL in the surgery and SBRT group for the other measured SF-36 scales, except for physical functioning and general health (Fig. 4). Recurrence rates and death rates are illustrated in Table 3.

**Table 3:** Recurrence rate of patients treated surgically or with SBRT. Four patients had both loco-regional recurrence and distant recurrence

	Surgery (%)	Radiotherapy (%)
All recurrence	9 (16)	3 (10)
Time till all recurrence(mean ± SD)	1.1 ± 0.7 months	0.4 ± 0.06 months
Local recurrence	1 (2)	–
Loco-regional recurrence	4 (7)	1 (3)
Distant recurrence	9 (16)	2 (7)
Death	5 (9)	8 (28)

#### SDM and HRQoL at baseline

No significant association could be found between HRQoL and patient experience with involvement in treatment selection (PCS *p*-value = 0.398, MCS *p*-value = 0.341), patient preferences for SDM (PCS *p*-values = 0.439, MCS *p*-value = 0.580), and final decision in lung cancer treatment selection (PCS *p*-value = 0.402, MCS *p*-value = 0.662).

## Discussion

This study illustrate that in the current clinical practice lung cancer patients experience decisional conflict and suboptimal information provision regarding the treatment and survival which highlights the need of improvement of information conveyance, and involvement of patients with early-stage NSCLC in treatment decision-making.

### Perceived patient knowledge regarding the treatment and communication with the patient

Up to one-fifth of patients reported lack of knowledge about the advantages and disadvantages of the treatment options and one-tenth of patients reported lack of knowledge about the treatment risks. These results illustrate that providing information needs to improve, particularly in an early stage of diagnosis and treatment because lung cancer patients are emotionally unstable and could be overloaded with information about their disease [39]. Numerous studies explored different strategies to improve and adopt SDM in clinical practice [40]. One of the main topics of improving cancer communication is ‘health literacy’ which involves the ability of the patient to read, understand, and use health information to make an appropriate decision. In order to achieve an effective communication it is essential to describe health state in language that is accessible to the patient and discuss the benefits and risks of treatment options in a balanced way [41, 42]. In the field of breast cancer it is illustrated that by deciding on a cancer treatment without fully understanding the associated risks and benefits could lead to overuse or underuse of cancer treatments [43, 44].

Additionally, the majority of patients felt sufficiently involved in treatment decision-making and indicated that they had enough time to make an informed decision. It was interesting to see that the minority of patients reported to have received information on survival. It is crucial to discuss survival and prognosis with the patient in a way that the patient will understand this information because previous studies have shown that the cancer patients overestimate their life expectancy and probabilities of cure when compared to their physicians’ perspective [45–47]. This will lead to unrealistic high expectations about the medical treatment which is a common phenomenon in oncology patients [48, 49].

### Experience with current clinical decision-making

The majority of patients had a strong desire to participate in treatment decision-making and preferred the decision to be the outcome of a SDM-process. This is in line with the previous studies showing that more patients preferred to participate rather than delegate decisions [50]. One of the challenges of SDM is knowing how much involvement a patient wants and needs. It is even more difficult when patients vary in the amount of control that they prefer to have over the treatment decision-making at the time of diagnosis [26]. Using tools such as decision aids prior to the consultation or during the visit will improve the communication between the patient and physician and there will be more time for the patient to absorb health care information and ask questions during the consultation [51, 52].

Forty percent of patients experienced decisional conflict, and one in five patients to such an extent that it made them feel unsure about the decision. Decisional conflict was most evident in the uncertainty and informed subscale, suggesting that improvement of patient uncertainty and better informing the patient before the treatment will improve the quality of decision-making [27]. The same rates has been reported by patients treated for other type of cancer [53, 54]. Various factors can play a role in high levels of decisional conflict in cancer patients. First, most cancer patients want as much information as possible, however, they could be overloaded with information when it is offered ‘all at once’ or when the information is not provided to the patients’ family [55]. As we have illustrated in this study, an inadequate level of perceived information contributes the most to decisional conflict. Second, periodic assessment of cancer patient’s information requirements is also crucial, considering the complexity of cancer care. Finally, in our previous study we have illustrated that patients who receive SBRT differ significantly from the surgical patients [56]. It is important to appreciate these differences and realize that SBRT patients do not always have a choice between treatment options.

Although decisional conflict is about what patients go through when confronted with a difficult decision, the idea of decisional conflict is also to help patients to think about participation in decision-making and motivate them to engage in treatment decision-making [57]. Furthermore, these scales also illustrate how patients are informed and where the improvements are needed.

### Health related quality of life and shared decision making

In general, lung cancer patients have poor HRQoL compared to the general population or patients without lung cancer [58, 59]. In this study, patients in the SBRT group scored at baseline lower on physical component summary compared to patients treated surgically. No differences could be found regarding the mental component summary. An explanation for the observed differences in HRQoL between the two groups could be the significant differences in baseline characteristics [2, 56]. No association could be found between HRQoL and different aspect of SDM meaning that in this study HRQoL was not positively or negatively influenced by patient experiences with SDM. Our findings are comparable with a number of studies concluding that there is weak evidence that aspects of SDM are positively or negatively associated with QoL outcomes [60].

### Strengths and limitations

The present study is a prospective observational cohort study allowing for new insights into the process of SDM and information conveyance in lung cancer patients. Although many articles have been written on SDM and patient participation in treatment decision-making in cancer patients, to our knowledge little research has been done on the role of early-stage lung cancer patients -treated surgically or with SBRT- in treatment decision-making and patients experiences and preferences regarding SDM. Also, the lung cancer patients were surveyed after diagnosis but before the treatment which allow us to investigate the unbiased perception of the patient regarding the treatment decision-making.

Potential limitations need to be addressed regarding the present study. First, the conceptual design of this study was not built on a specific theory. We explicitly chose to include all patients with stage I or II NSCLC who were planned for a surgical treatment or SBRT. We wanted to illustrate the patient participation in treatment decision-making, since there is little research about the role of early-stage lung cancer patients -treated surgically or with SBRT- in treatment decision-making. Second, overall response rate was 54% thus making the sample size of this study small. The non-responders were contacted to ask why they would not be part of the study. The following major reasons were given: 1) they were shocked by the diagnosis and therefore they did not want to complete the questionnaire; 2) they were too preoccupied with their illness and therefore they had no time for the questionnaire; 3) the questionnaire was too confrontational. However, no significant differences were found between responders and non-responders in terms of baseline characteristics. Third, we are aware of the shortcomings of using GLM. By using the bootstrap method we have tried to account for this inadequacy. However, no differences were observed between the results of GLM and results of GLM with bootstrapping. Finally, the response rate at 6 and 12 months was low due to recurrences rates and death rates in both treatment groups making analyses of HRQoL at 6 and 12 months difficult.

## Conclusions

Shared-decision-making (SDM), where patients are involved as active partners with the physician in treatment decisions, is an important part of patient-centered cancer care as it weighs the pros and cons of treatment options while taking patients values and preferences into account.

Dutch early-stage NSCLC patients find it important to be involved in treatment decision-making. The majority of patients in this study found it important to be involved in decision-making and reported that they felt sufficiently involved by their treating physician. Yet a substantial proportion of patients experiences decisional conflict and feels uninformed. HRQoL was not influenced by patient experiences with SDM. Better patient information, and patient involvement in treatment decision-making is needed in order to improve patient knowledge and hopefully reduce decisional conflict.

## Additional file

Additional file 1: Questionnaire used in the study. Description of data: Questionnaire used in the study. (DOC 50 kb)

## Abbreviations

ACCP: American College of Chest Physicians
CCI: Charlson-Comorbidity-Index
COPD: Chronic obstructive pulmonary disease
CPS: Control Preferences Scale
DCS: Decisional Conflict Scale
GLM: General linear model
HRQoL: Health related quality of life
MCS: Mental component summary
NSCLC: Non-small cell lung cancer
PCS: Physical component summary
SBRT: Stereotactic body radiotherapy
SDM: Shared-decision-making
SF-36: Short-Form 36-Item Health Survey
STS: Society of Thoracic Surgeons
