# Supplementary material for: Treatment selection of early stage non-small cell lung cancer: the role of the patient in clinical decision making
Source: BMC Cancer. 2018 Jan 15;18:79. doi: 10.1186/s12885-018-3986-5 (PMC5769349; doi:10.1186/s12885-018-3986-5)
Supplement: Additional file 1: — Questionnaire used in the study. Description of data: Questionnaire used in the study. (DOC 50 kb) [file 12885_2018_3986_MOESM1_ESM.doc]

Questionnaire used in the study

1. What is your name?

……………………………………………………………………………………………….........................

2. What is your gender?

- Male
- Female

3. What is your date of birth?

……………………………………………………………………………………………….........................

4. What is the highest level of education you have completed?

- Primary school
- Secondary school, level:…………………………………………………………………………….
- Higher education, namely:………………………………………………………………………….
- University, degree:………………………………………………………………………………….
- I would rather not say

5. In which hospital are you being treated?

……………………………………………………………………………………………………………….

6. Have you been referred from another hospital?

- Yes, namely………….……………………………………………………………………………
- No, I was immediately referred to this center by the general practitioner

7. By whom will you be treated?

- Lung surgeon/cardiothoracic surgeon
- Lung physician
- Radiotherapist
- Other, namely……………………………………………………………………………………….

8. With how many people did you have a conversation in the hospital about your treatment?

- 1
- 2
- 3
- 4
- More, namely……………………………………………………………………………………….

The following questions are about the conversation that you have had with your attending physician about different treatment options.

9. Has everything been addressed during the conversation about your treatment?

- Yes
- No, because…………………………………………………………………………………………

10. Please indicate below which topics are discussed during the conversation about your treatment (multiple answers are possible).

- General information
- Advantages and disadvantages of a treatment
- Risks of a treatment
- Expected adverse effects
- Duration of a treatment (in weeks, months or years)
- Duration of hospitalization
- Recovery period
- Survival
- And………………………………………………………………………………………………….

11. Do you think you have enough knowledge about the pros and the cons of the different treatment options?

Totally agree 1 2 3 4 5 Totally disagree

12. Do you think you are adequately aware of the risks of your treatment?

- Yes
- No, because…………………………………………………………………………………………

13. Do you think you are adequately involved in the decision for a treatment?

Totally agree 1 2 3 4 5 Totally disagree

The following questions are about the decision-making process regarding the choice for a particular treatment for lung cancer. By answering these questions, you will indicate your opinion regarding this decision-making process.

14. With who did you have the conversation about the choice for a particular treatment (multiple answers possible)?

- With the lung surgeon/cardiothoracic surgeon
- With the lung physician
- With the radiotherapist
- Other, namely……………………………………………………………………………………….

15. What was the date of this conversation?

………………………………………………………………………………………………….....................

16. Have you had enough time to make an informed decision?

- Yes
- No
- I do not know
- I have not taken a decision myself

17. Has anyone else been involved in making the choice for a particular treatment?

- Yes, a family member
- Yes, a good friend
- Yes,…………………………………………………………………………………………………
- No,…………………………………………………………………………………………………..

Below are two statements regarding the treatment choice, you can indicate to what extent you agree with the statement by encircling a grade; 1=totally agree, 5=totally disagree. If the question does not apply to your situation or you do not know the answer you can indicate this.

18. The physician has involved me in making a choice for a particular treatment for lung cancer.

Totally agree 1 2 3 4 5 Totally disagree

- I do not know
- Not applicable

19. I think it is important to be involved in choosing a treatment

Totally agree 1 2 3 4 5 Totally disagree

20. Do you feel that you have had a choice in receiving a particular treatment?

- Yes
- No
- I do not know

Explanation:…………………………………………………………………………………………………………………………………………………………………………………………………………………………………………………………………………………………………………………………………

21. The final choice for a particular treatment for lung cancer should be done by:

- Physician
- Physician, after patient opinion
- Physician and patient
- Patient, after physician opinion
- Patient

**Decision conflict scale**

Decision conflict scale was explored by using the Dutch version of Decisional Conflict Scale.

**Health related quality of life**

Health related quality of life was explored by using SF-36 questionnaire.

Details regarding questionnaires of decision conflict scale and health related quality of life are described in the method section with references.
